# Risk factors for Charcot foot development in individuals with diabetes mellitus

**DOI:** 10.1007/s00125-024-06271-9

**Published:** 2024-09-13

**Authors:** Georgios Tsatsaris, Neda Rajamand Ekberg, Tove Fall, Sergiu-Bogdan Catrina

**Affiliations:** 1 Department of Molecular Medicine and Surgery, Karolinska Institutet, Stockholm, Sweden; 2 Centre for Diabetes, Academic Specialist Centrum, Stockholm, Sweden; 3 Department of Endocrinology and Diabetes, Karolinska University Hospital, Stockholm, Sweden; 4 Department of Medical Sciences, Molecular Epidemiology and Science for Life Laboratory, Uppsala University, Uppsala, Sweden

**Keywords:** Charcot foot, Charcot osteoarthropathy, Diabetes, Diabetes complications, Risk factors

## Abstract

**Aims/hypothesis:**

Charcot foot is a complication of diabetes mellitus that has potentially disastrous consequences. Although it was first described in 1868 and found to be associated with diabetes in 1936, there is still uncertainty about the risk factors affecting the development of the condition. Here, we aim to identify risk factors for Charcot foot in a nationwide cohort study.

**Methods:**

A retrospective register-based cohort study was performed for the period 2001–2016, using nationwide registries. Individuals with diabetes and Charcot foot were identified and matched by diabetes type and with similar diabetes duration with individuals with diabetes but not Charcot foot. Logistic regression analyses were used to identify risk factors.

**Results:**

A total of 3397 participants with diabetes mellitus and Charcot foot and 27,662 control participants with diabetes but without Charcot foot were included. HbA_1c_, duration of diabetes, micro- and macroalbuminuria, retinopathy and atherosclerosis (general and peripheral) were identified as risk factors for Charcot foot in participants with type 1 diabetes and participants with type 2 diabetes.

**Conclusions/interpretation:**

In the most extensive study on Charcot foot to date, we identified distinctive and common risk factors associated with the development of Charcot foot in individuals with type 1 diabetes and type 2 diabetes.

**Graphical Abstract:**

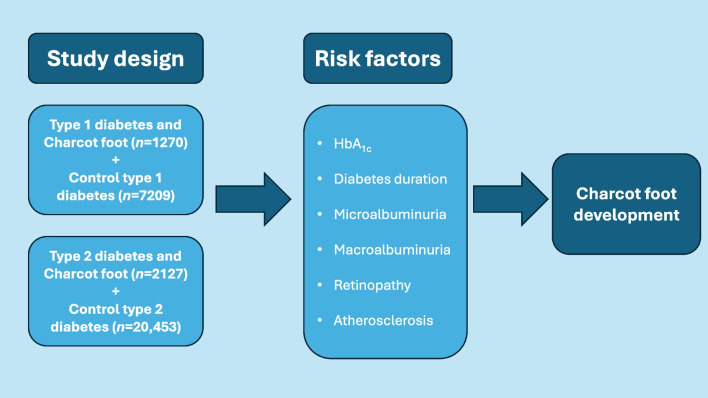

**Supplementary Information:**

The online version of this article (10.1007/s00125-024-06271-9) contains peer-reviewed but unedited supplementary material.


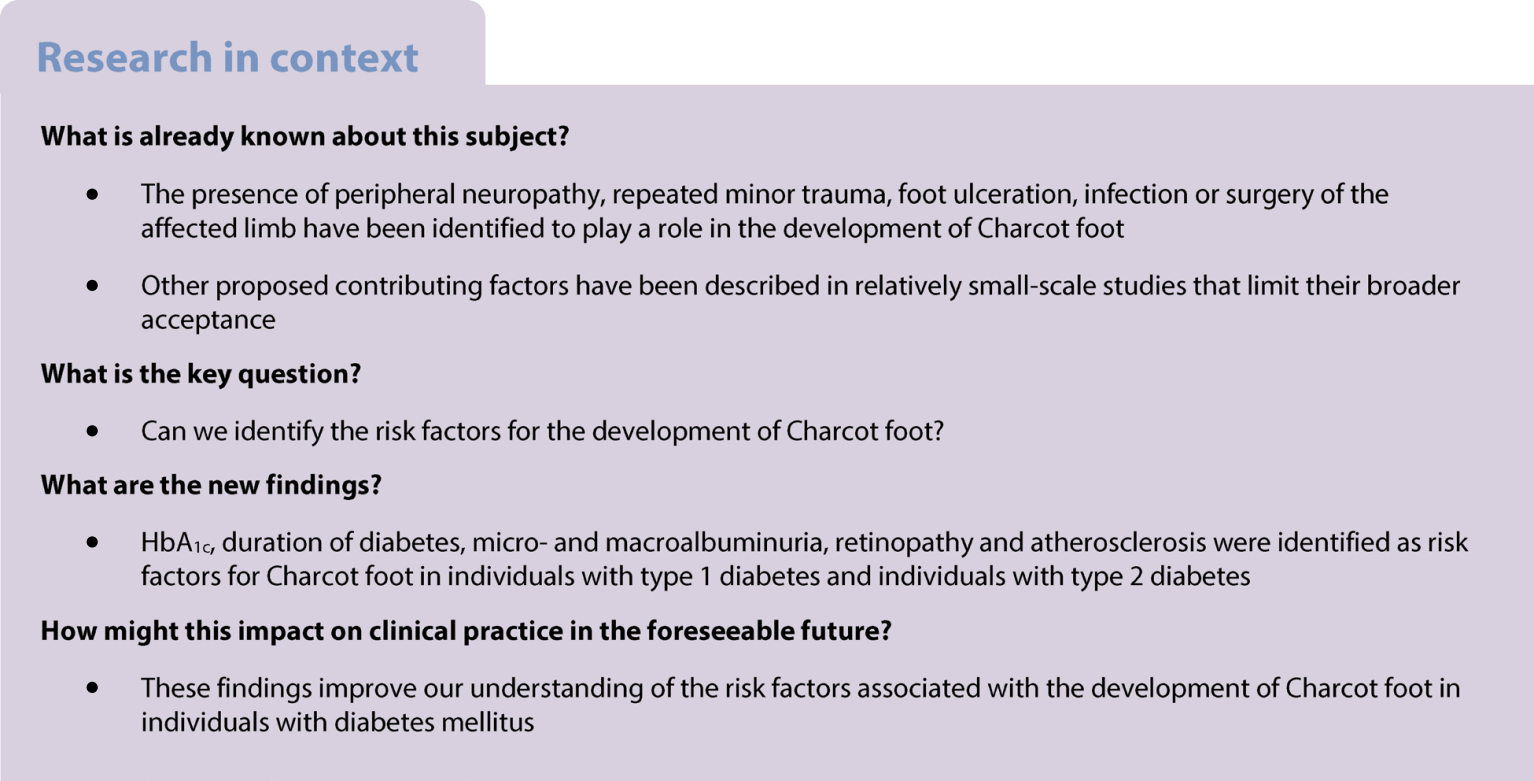


## Introduction

Charcot osteoarthropathy, commonly called Charcot foot, is a severe complication of diabetes mellitus with potential disastrous consequences. Although Jean Martin Charcot initially described neuroarthropathic joint changes in patients with tabes dorsalis in 1868 [1], it was William Riely Jordan who identified the association between Charcot osteoarthropathy with diabetes mellitus in 1936 [2]. Because the prevalence of the neurological complications of syphilis has substantially decreased, diabetes is now the predominant cause of Charcot foot [3]. Charcot foot is an inflammatory condition that affects the soft tissues and bones of the foot and the ankle joints and can lead to bone fractures, dislocations and joint destruction [4].

The diagnosis of Charcot foot is challenging [5] and is reported to be delayed in up to 25% of affected individuals [6]. If left untreated, the condition progresses, leading to substantial deformities of the foot [5–8] that increase the risks of chronic foot ulcers and lower extremity amputation (by 15- to 40-fold) [9]. Recently, two large register-based studies in Scandinavia reported a prevalence of 0.56–0.79%, with an incidence rate of 6.4–9.5 per 10,000 with diabetes [10, 11]. There is therefore a need to identify risk factors and establish a robust framework for forecasting the development of Charcot foot with the aim of improving early diagnosis and treatment for better outcomes.

The pathogenesis of Charcot foot in individuals with diabetes mellitus remains to be fully characterised but several factors in addition to the presence of peripheral neuropathy have been identified, including repeated minor trauma, foot ulceration, infection or surgery of the affected limb [12–14]. Small studies have suggested that HbA_1c_, presence of other diabetic complications and longer duration of diabetes are risk factors [12, 15–20] whereas peripheral arterial disease has been suggested to be protective [18]. However, the limited size of these studies affects their generalisability.

Robust data on risk factors for Charcot foot are crucial in clinical practice for designing effective healthcare strategies and developing new strategies for prevention and treatment of Charcot foot. Using databases provided by Swedish nationwide registries, we have conducted to the best of our knowledge the most extensive observational study of individuals with Charcot foot to date.

## Methods

### Study design and data sources

We conducted an observational study of data from two nationwide Swedish registries with the aim of identifying risk factors for development of Charcot foot in individuals with diabetes mellitus. The National Diabetes Register (NDR) was initiated in 1996, with a continuously improved coverage reaching up to 87.7% in 2020 throughout Sweden [11] for individuals aged ≥18 years who have been diagnosed with diabetes mellitus. It includes demographic, clinical and laboratory data and lists type of medication, as recorded by a healthcare provider. All included individuals have agreed to inclusion in the register through informed consent. The National Patient Register (NPR) contains data in the form of ICD-10 codes (https://icd.who.int/browse10/2019/en), which are associated with diagnoses related to hospitalisations and outpatient visits. Cross-links between these two registries were established with the help of the personal identification number, which is unique for every Swedish resident. Data was anonymised before delivery to the researchers.

The study was approved by the Regional Ethics Review Board in Stockholm (diary approval numbers 2016/652–31/1, 2016/1429–32, 2016/2141–32 and 2017/410–32).

### Study participants

All individuals with diabetes mellitus registered in the NDR, aged ≥18 years, were included in the study. Diabetes mellitus was defined using an inpatient or outpatient visit with the use of an appropriate reported ICD-10 code (E10–E14). Type 1 diabetes was defined using the ICD-10 code E10 and type 2 diabetes was defined using the ICD-10 code E11. The exclusive use of insulin did not classify a participant as having type 1 diabetes. If there was a discrepancy regarding the recorded diagnostic codes for the types of diabetes, the most recent record was assumed to be the most accurate. Charcot foot was defined using an inpatient or outpatient diagnosis of ICD-10 code M14.2, M14.6 or M90.8 in the NPR. The date of diagnosis of diabetes was recorded as the date of the initial diagnosis.

### Risk factors

Individuals with diabetes mellitus but no Charcot foot who were registered in the NDR (during the study period) were matched with the highest available number of control individuals (7:1 for those with type 1 diabetes and Charcot foot; 10:1 for those with type 2 diabetes and Charcot foot), with respect to the length of time (similar number of years of diabetes duration) they had had the disease at time of initial diagnosis of Charcot foot (index date). Potential risk factors for Charcot foot were identified from NDR and NPR data. Data regarding demographics, clinical characteristics, biochemical variables and therapies for blood glucose, lipid and BP regulation were identified in the NDR (from January 2001 until December 2016). Data collected for the 12 months preceding the index date was used.

In the NDR, HbA_1c_ values are reported dually as mmol/mol (International Federation of Clinical Chemistry and Laboratory Medicine) and % (National Glycohemoglobin Standardization Program). Microalbuminuria was defined using an albumin/creatinine ratio of 3–30 mg/mmol or a urine albumin concentration of 20–200 µg/min; macroalbuminuria was defined using an albumin/creatinine ratio of >30 mg/mmol or a urine albumin concentration of >200 µg/min. Two positive tests obtained from at least three different samples within one calendar year were needed to confirm this finding, otherwise individuals were defined as not having albuminuria. The presence of retinopathy was reported according to the existing national guidelines [21] and defined using the presence of alterations or damage to the retina caused by diabetes mellitus. BMI was defined as low/normal (≤25 kg/m^2^) or high (>25 kg/m^2^). Hypertension was defined as a BP of ≥140/85 mmHg. While both main and secondary diagnoses of peripheral vascular disease (PVD) (ICD-9 code [http://www.icd9data.com/2007/Volume1/default.htm] 443.9; ICD-10 code I73), atherosclerosis (ICD-9 code 440; ICD-10 code I70) and osteoporosis (ICD-9 code 733; ICD-10 code M81) were identified in the NPR, diagnoses of prior ischaemic heart disease or stroke, along with the use of acetylsalicylic acid and BP- and lipid-lowering medication were identified in the NDR.

### Statistical analysis

#### Descriptive data

The cohorts were divided into two independent groups according to the type of diabetes. Data are described using means ± SDs for normally distributed numeric data, and medians (IQRs) for data with skewed distributions. Counts and percentages are presented for categorical data. Two-sample *t* tests were used to compare mean values, Pearson’s χ^2^ test was used to test the difference between proportions and Wilcoxon rank-sum test was used to compare distributions of skewed distributed variables.

#### Potential causal links

To facilitate the evaluation of potential causal links between HbA_1c_, duration of diabetes, macroalbuminuria, microalbuminuria, atherosclerosis and retinopathy and Charcot foot, we created direct acyclic graphs (DAGs) to identify possible confounders for inclusion in the statistical analysis.

Applying d-separation criteria on the DAG, the following terms were indicated as confounders: for HbA_1c_, we used age, macroalbuminuria, microalbuminuria (type 1 diabetes and type 2 diabetes) and atherosclerosis (type 2 diabetes) (ESM Figs 1, 2) [22, 23]; for duration of diabetes, we used age (type 1 diabetes and type 2 diabetes) (ESM Fig. 3); for macroalbuminuria, we used age, HbA_1c_ and BP (type 1 diabetes and type 2 diabetes); for microalbuminuria, we used age, HbA_1c_ and BP (type 1 diabetes and type 2 diabetes) (ESM Fig. 4); for retinopathy, we used age, LDL-cholesterol, plasma triglycerides, HbA_1c_ and BP (type 1 diabetes and type 2 diabetes) (ESM Fig. 5); and for atherosclerosis, we used age, HbA_1c_, LDL-cholesterol, HDL-cholesterol, triglycerides, duration of diabetes, BP, BMI and smoking (type 1 diabetes and type 2 diabetes) (ESM Fig. 6).

In addition, a sensitivity analysis was performed by reproducing the logistic regression models with the category ‘unknown’ included for BMI, BP, creatinine and diabetic retinopathy. This category was introduced to increase statistical power.

The robust sandwich estimator was used to estimate SEs and two-sided *p* values are reported. A *p* value of <0.05 was considered to represent statistical significance. The analyses were performed using Stata version 15 (StataCorp, College Station, TX, USA) and were conducted at the Biostatistics Core Facility of the Karolinska Institutet in Stockholm.

## Results

**Fig. 1:**
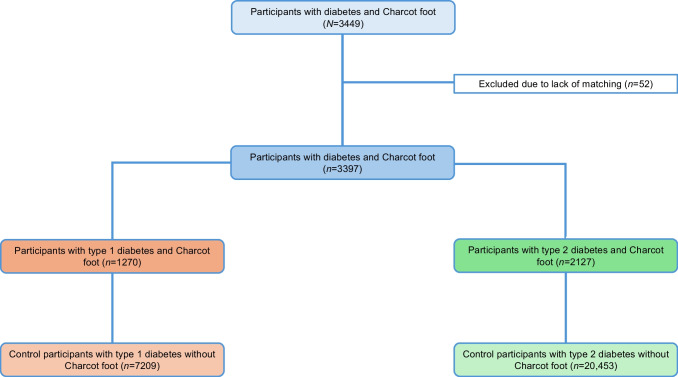
Flow chart showing study design and inclusion of participants

A total of 3449 individuals with diabetes mellitus and Charcot foot were initially identified. Fifty-two were excluded from the study due to lack of matching. Participants with a specific type of diabetes were matched by the duration of diabetes with participants from the total diabetic population without Charcot foot. In the end, 3397 diabetic participants (98.5%) (1270 with type 1 diabetes, 2127 with type 2 diabetes), along with 27,662 control participants, were finally included in the study. A detailed flow chart of the study design is provided in Fig. 1.

Participants with type 1 diabetes and Charcot foot had lower mean age, BMI, BP (both systolic and diastolic), lower percentage of ischaemic episodes and atherosclerosis and longer duration of diabetes but had higher HbA_1c_ and a higher incidence of retinopathy compared with participants with type 2 diabetes and Charcot foot. Women with type 1 diabetes (50.4%) and men with type 2 diabetes (68.1%) developed Charcot foot at higher rates than their counterparts of the opposite sex (Table 1 and ESM Table 1).

**Table 1:** Characteristics of the participants with type 1 and type 2 diabetes and Charcot foot

Characteristic	Type 1 diabetes (*N*=1270)	Type 2 diabetes (*N*=2127)	All (*N*=3397)
Age, years	51.77 ± 11.80	64.50 ± 10.87	59.74 ± 12.80
Sex			
Male	630 (49.6)	1448 (68.1)	2078 (61.2)
Female	640 (50.4)	679 (31.9)	1319 (38.8)
Diabetes duration, years	32.73 ± 12.97	14.59 ± 9.41	21.37 ± 13.98
Weight, kg	78.98 ± 16.81	95.48 ± 20.10	89.11 ± 20.53
Height, cm	172.88 ± 9.83	174.98 ± 10.53	174.17 ± 10.31
BMI, kg/m^2^	26.28 ± 4.49	31.02 ± 5.54	29.17 ± 5.65
BMI category			
Low/normal	507 (39.9)	233 (11.0)	740 (21.8)
High	678 (53.4)	1627 (76.5)	2305 (67.9)
Unknown	85 (6.7)	267 (12.6)	352 (10.4)
Systolic BP, mmHg	133.29 ± 18.89	138.30 ± 18.45	136.40 ± 18.77
Diastolic BP, mmHg	73.45 ± 10.46	76.53 ± 10.56	75.36 ± 10.63
BP category			
Low/normal	1109 (87.3)	1668 (78.4)	2777 (81.7)
High	133 (10.5)	364 (17.1)	497 (14.6)
Unknown	28 (2.2)	95 (4.5)	123 (3.6)
HbA_1c_, mmol/mol	68.00 (58.50–78.00)	58.00 (49.00–71.00)	63.00 (52.00–74.00)
HbA_1c_, %	8.4 (7.5–9.3)	7.5 (6.6–8.6)	7.9 (6.9–8.9)
Cholesterol, mmol/l	4.73 ± 1.06	4.67 ± 1.16	4.70 ± 1.12
HDL-cholesterol, mmol/l	1.50 (1.20–1.90)	1.10 (0.90–1.40)	1.20 (1.00–1.60)
Triglycerides, mmol/l	1.10 (0.80–1.60)	1.80 (1.20–2.70)	1.50 (1.00–2.20)
LDL-cholesterol, mmol/l	2.45 (1.97–2.98)	2.43 (1.84–3.11)	2.44 (1.90–3.05)
Creatinine, μmol/l	83.00 (68.00–113.00)	84.00 (69.00–109.00)	84.00 (69.00–110.00)
Macroalbuminuria	270 (21.3)	361 (17.0)	631 (18.6)
Microalbuminuria	352 (27.7)	559 (26.3)	911 (26.8)
BP medication	850 (66.9)	1598 (75.1)	2448 (72.1)
Lipid-lowering medication	652 (56.5)	1147 (60.1)	1799 (58.7)
Previous osteoporosis			
No	1235 (97.2)	2109 (99.2)	3344 (98.4)
Yes	35 (2.8)	18 (0.8)	53 (1.6)
Diseases of arteries			
No	1173 (92.4)	1908 (89.7)	3081 (90.7)
Yes	97 (7.6)	219 (10.3)	316 (9.3)
Previous PVD			
No	1177 (92.7)	1915 (90.0)	3092 (91.0)
Yes	93 (7.3)	212 (10.0)	305 (9.0)
Previous atherosclerosis			
No	1184 (93.2)	1928 (90.6)	3112 (91.6)
Yes	86 (6.8)	199 (9.4)	285 (8.4)
Previous atherosclerosis in extremity			
No	1188 (93.5)	1936 (91.0)	3124 (92.0)
Yes	82 (6.5)	191 (9.0)	273 (8.0)
Acetylsalicylic acid use	433 (34.1)	867 (40.8)	1300 (38.3)
Previous ischaemic heart disease	191 (15.0)	462 (21.7)	653 (19.2)
Previous stroke	63 (5.0)	159 (7.5)	222 (6.5)
Diabetic retinopathy			
None	77 (6.1)	462 (21.7)	539 (15.9)
Retinopathy	805 (63.4)	747 (35.1)	1552 (45.7)
Unknown	388 (30.6)	918 (43.2)	1306 (38.4)

Continuous data are presented as mean ± SD or median (IQR), and categorical data are presented as *n* (%)

Two-sample *t* tests were used to compare mean values, Pearson’s χ^2^ test was used to test the difference between proportions and Wilcoxon rank-sum test was used to compare distributions of skewed distributed variables

PVD, peripheral vascular disease

Participants with type 1 diabetes and Charcot foot exhibited a higher prevalence of retinopathy, micro- and macroalbuminuria, atherosclerosis and elevated HbA_1c_ (68 mmol/mol vs 64 mmol/mol) than the control group of participants with type 1 diabetes but without Charcot foot (Table 2 and ESM Table 2). Similarly, those with type 2 diabetes and CF also demonstrated these conditions more frequently than their respective control group of participants with type 2 diabetes but without CF (Table 3 and ESM Table 3).

**Table 2:** Characteristics of the matched participants with type 1 diabetes with or without Charcot foot

Characteristic	Type 1 diabetes without Charcot foot (*N*=7209)	Type 1 diabetes with Charcot foot (*N*=1270)	All (*N*=8479)
Age, years	46.97 ± 17.80	51.77 ± 11.80	47.69 ± 17.12
Sex			
Male	4031 (55.9)	630 (49.6)	4661 (55.0)
Female	3178 (44.1)	640 (50.4)	3818 (45.0)
Diabetes duration, years	27.61 ± 12.37	32.73 ± 12.97	28.38 ± 12.59
BMI, kg/m^2^	25.53 ± 4.19	26.28 ± 4.49	25.66 ± 4.25
BMI category			
Low/normal	2889 (40.1)	507 (39.9)	3396 (40.1)
High	2842 (39.4)	678 (53.4)	3520 (41.5)
Unknown	1478 (20.5)	85 (6.7)	1563 (18.4)
BP			
Low/normal	6004 (83.3)	1109 (87.3)	7113 (83.9)
High	595 (8.3)	133 (10.5)	728 (8.6)
Unknown	610 (8.5)	28 (2.2)	638 (7.5)
HbA_1c_, mmol/mol	64.00 (55.00–73.00)	68.00 (58.50–78.00)	65.00 (56.00–74.00)
HbA_1c_, %	8.0 (7.2–8.8)	8.4 (7.5–9.3)	8.1 (7.3–8.9)
Cholesterol, mmol/l	4.87 ± 1.02	4.73 ± 1.06	4.84 ± 1.03
HDL-cholesterol, mmol/l	1.60 (1.30–1.90)	1.50 (1.20–1.90)	1.60 (1.30–1.90)
Triglycerides, mmol/l	1.00 (0.70–1.40)	1.10 (0.80–1.60)	1.00 (0.70–1.40)
LDL-cholesterol, mmol/l	2.64 (2.12–3.22)	2.45 (1.97–2.98)	2.60 (2.10–3.18)
Creatinine, μmol/l	76.00 (65.00–89.00)	83.00 (68.00–113.00)	77.00 (65.00–91.00)
Macroalbuminuria	500 (6.9)	270 (21.3)	770 (9.1)
Microalbuminuria	882 (12.2)	352 (27.7)	1234 (14.6)
BP medication	2926 (40.6)	850 (66.9)	3776 (44.5)
Lipid-lowering medication	2148 (32.7)	652 (56.5)	2800 (36.3)
Previous osteoporosis			
No	7173 (99.5)	1235 (97.2)	8408 (99.2)
Yes	36 (0.5)	35 (2.8)	71 (0.8)
Diseases of arteries			
No	7067 (98.0)	1173 (92.4)	8240 (97.2)
Yes	142 (2.0)	97 (7.6)	239 (2.8)
Previous PVD			
No	7074 (98.1)	1177 (92.7)	8251 (97.3)
Yes	135 (1.9)	93 (7.3)	228 (2.7)
Previous atherosclerosis			
No	7100 (98.5)	1184 (93.2)	8284 (97.7)
Yes	109 (1.5)	86 (6.8)	195 (2.3)
Previous atherosclerosis in extremity			
No	7107 (98.6)	1188 (93.5)	8295 (97.8)
Yes	102 (1.4)	82 (6.5)	184 (2.2)
Acetylsalicylic acid	1347 (18.7)	433 (34.1)	1780 (21.0)
Previous ischaemic heart disease	580 (8.0)	191 (15.0)	771 (9.1)
Previous stroke	233 (3.2)	63 (5.0)	296 (3.5)
Diabetic retinopathy			
None	1302 (18.1)	77 (6.1)	1379 (16.3)
Retinopathy	2629 (36.5)	805 (63.4)	3434 (40.5)
Unknown	3278 (45.5)	388 (30.6)	3666 (43.2)
Smoking			
No	5173 (71.8)	896 (70.6)	6069 (71.6)
Yes	903 (12.5)	126 (9.9)	1029 (12.1)
Unknown	1133 (15.7)	248 (19.5)	1381 (16.3)

Continuous data are presented as mean ± SD or median (IQR), and categorical data are presented as *n* (%)

Two-sample *t* tests were used to compare mean values, Pearson’s χ^2^ test was sued to test the difference between proportions and Wilcoxon rank-sum test was used to compare distributions of skewed distributed variables

PVD, peripheral vascular disease

**Table 3:** Characteristics of the matched participants with type 2 diabetes with or without Charcot foot

Characteristic	Type 2 diabetes without Charcot foot (*N*=20,453)	Type 2 diabetes with Charcot foot (*N*=2127)	All (*N*=22,580)
Age, years	68.01 ± 11.55	64.50 ± 10.87	67.68 ± 11.53
Sex			
Male	11,477 (56.1)	1448 (68.1)	12,925 (57.2)
Female	8976 (43.9)	679 (31.9)	9655 (42.8)
Diabetes duration, years	14.04 ± 8.68	14.59 ± 9.41	14.09 ± 8.75
BMI, kg/m^2^	29.55 ± 5.33	31.02 ± 5.54	29.70 ± 5.37
BMI category			
Low/normal	3216 (15.7)	233 (11.0)	3449 (15.3)
High	13804 (67.5)	1627 (76.5)	15431 (68.3)
Unknown	3433 (16.8)	267 (12.6)	3700 (16.4)
BP category			
Low/normal	15977 (78.1)	1668 (78.4)	17645 (78.1)
High	2905 (14.2)	364 (17.1)	3269 (14.5)
Unknown	1571 (7.7)	95 (4.5)	1666 (7.4)
HbA_1c_, mmol/mol	55.00 (48.00–65.00)	58.00 (49.00–71.00)	55.00 (48.00–66.00)
HbA_1c_, %	7.2 (6.5–8.1)	7.5 (6.6–8.6)	7.2 (6.5–8.2)
Cholesterol, mmol/l	4.75 ± 1.11	4.67 ± 1.16	4.74 ± 1.11
HDL-cholesterol, mmol/l	1.20 (1.00–1.50)	1.10 (0.90–1.40)	1.20 (1.00–1.40)
Triglycerides, mmol/l	1.60 (1.10–2.20)	1.80 (1.20–2.70)	1.60 (1.20–2.20)
LDL-cholesterol, mmol/l	2.55 (2.00–3.23)	2.43 (1.84–3.11)	2.54 (1.98–3.21)
Creatinine, μmol/l	78.00 (65.00–94.00)	84.00 (69.00–109.00)	78.00 (66.00–95.00)
Macroalbuminuria	1500 (7.3)	361 (17.0)	1861 (8.2)
Microalbuminuria	3214 (15.7)	559 (26.3)	3773 (16.7)
BP medication	14,709 (71.9)	1598 (75.1)	16,307 (72.2)
Lipid-lowering medication	10,744 (57.4)	1147 (60.1)	11,891 (57.7)
Previous osteoporosis			
No	20,336 (99.4)	2109 (99.2)	22,445 (99.4)
Yes	117 (0.6)	18 (0.8)	135 (0.6)
Diseases of arteries			
No	20,022 (97.9)	1908 (89.7)	21,930 (97.1)
Yes	431 (2.1)	219 (10.3)	650 (2.9)
Previous PVD			
No	20,076 (98.2)	1915 (90.0)	21,991 (97.4)
Yes	377 (1.8)	212 (10.0)	589 (2.6)
Previous atherosclerosis			
No	20,115 (98.3)	1928 (90.6)	22,043 (97.6)
Yes	338 (1.7)	199 (9.4)	537 (2.4)
Previous atherosclerosis in extremity			
No	20,174 (98.6)	1936 (91.0)	22,110 (97.9)
Yes	279 (1.4)	191 (9.0)	470 (2.1)
Acetylsalicylic acid	7972 (39.0)	867 (40.8)	8839 (39.1)
Previous ischaemic heart disease	4394 (21.5)	462 (21.7)	4856 (21.5)
Previous stroke	1644 (8.0)	159 (7.5)	1803 (8.0)
Diabetic retinopathy			
None	6719 (32.9)	462 (21.7)	7181 (31.8)
Retinopathy	4176 (20.4)	747 (35.1)	4923 (21.8)
Unknown	9558 (46.7)	918 (43.2)	10476 (46.4)
Smoking			
No	13,020 (63.7)	1310 (61.6)	14,330 (63.5)
Yes	2187 (10.7)	204 (9.6)	2391 (10.6)
Unknown	5246 (25.6)	613 (28.8)	5859 (25.9)

Continuous data are presented as mean ± SD or median (IQR), and categorical data are presented as *n* (%)

Two-sample *t* tests were used to compare mean values, Pearson’s χ^2^ test was sued to test the difference between proportions and Wilcoxon rank-sum test was used to compare distributions of skewed distributed variables

PVD, peripheral vascular disease

Due to incomplete reports in the NDR database, it was not possible to evaluate foot risk grade as an individual variable.

### Potential causal risks

After using DAGs and adjusting for potential confounders, we performed logistic regression analysis to discover potential causal links between HbA_1c_, duration of diabetes, micro- and macroalbuminuria, retinopathy and atherosclerosis with the development of Charcot foot. Atherosclerosis was related to a higher risk of developing Charcot foot in both participants with type 1 diabetes (OR 3.32 [95% CI 2.27, 4.86]) and participants with type 2 diabetes (OR 8.60 [95% CI 6.79, 10.87]) (Table 4). The presence of diabetic complications (micro- and macroalbuminuria, retinopathy) was also associated with a higher risk for Charcot foot in both participants with type 1 diabetes and participants with type 2 diabetes, as were HbA_1c_ and duration of diabetes (Table 4).

**Table 4:** Results of logistic regression analysis after performing DAGs and adjusting for potential confounders

Risk factor	Type 1 diabetes		Type 2 diabetes	
	OR (95% CI)	*p* value	OR (95% CI)	*p* value
HbA_1c_	1.02 (1.01, 1.02)	<0.001	1.01 (1.01, 1.01)	<0.001
Duration of diabetes	1.03 (1.02, 1.03)	<0.001	1.02 (1.01, 1.02)	<0.001
Macroalbuminuria	3.12 (2.63, 3.71)	<0.001	2.54 (2.23, 2.89)	<0.001
Microalbuminuria	2.31 (2.00, 2.68)	<0.001	1.89 (1.70, 2.10)	<0.001
Retinopathy	4.10 (3.19, 5.43)	<0.001	2.66 (2.31, 3.05)	<0.001
Atherosclerosis	3.32 (2.27, 4.86)	<0.001	8.60 (6.79, 10.89)	<0.001

## Discussion

Charcot foot is a severe complication of diabetes that affects only a minority of individuals but carries a significant risk of delayed diagnosis [11]. Identifying the risk factors for its development is therefore crucial for focusing attention on at-risk individuals, allowing early diagnosis and treatment. Using data from 3397 cases of Charcot foot, the largest cohort of individuals with Charcot foot secondary to diabetes mellitus studied to date, we have identified both common and specific risk factors for individuals with type 1 diabetes and individuals with type 2 diabetes.

By using DAGs that identify possible confounders, we have separately examined potential causal links between HbA_1c_, duration of diabetes, micro- and macroalbuminuria, retinopathy, atherosclerosis and the development of Charcot foot. After analysing the DAGs, the most significant risk factors for Charcot foot differed between individuals with type 1 diabetes and individuals with type 2 diabetes (pre-existing microvascular complications and atherosclerosis, respectively). However, these variables were not adjusted for socioeconomic factors and sex, which may represent potential cofounders. While some of these risk factors have previously been associated with peripheral neuropathy, they have never been extensively examined in individuals with Charcot foot. Furthermore, we identified for the first time that the presence of atherosclerosis is a significant risk factor for the development of Charcot foot.

The main strength of the present study is the nationwide registry-based study design with wide coverage, including almost all patients with diabetes in Sweden, resulting in a large sample size with numerous biochemical and epidemiological data. However, there were some limitations. Although the matching was planned to be 1:10, in type 1 diabetes this was not possible, resulting in a smaller ratio of matching and causing a difference in the duration of diabetes. Data in the NDR are subject to bias through missing values. To minimise the risk of such bias, we verified results by performing a sensitivity analysis in which the missing values were included in an ‘unknown’ category; this resulted in similar conclusions to those presented. Additionally, the use of a broad definition for Charcot foot may lead to an overestimation of Charcot foot. To address this, we have contacted, and included the ICD codes used by, all university and other large regional hospitals in Sweden having a multidisciplinary team for diabetic foot ulcers. The same ICD codes were also used in another epidemiological study conducted in Denmark [10]. Finally, although peripheral neuropathy is inherently present in individuals with Charcot foot, we did not have any reliable data to evaluate this.

In conclusion, in this largest cohort of Charcot foot in individuals with diabetes, we have identified diabetes duration, retinopathy, micro- and macroalbuminuria, elevated HbA_1c_ levels and the presence of atherosclerosis as significant risk factors for development of Charcot foot within 12 months. Additionally, women with type 1 diabetes and men with type 2 diabetes have higher risk for developing Charcot foot compared with their counterparts of different sex. Similar studies in other populations are warranted to determine the generalisability of these results.

## Supplementary Information

Below is the link to the electronic supplementary material.

ESM (PDF 883 KB)

## Data Availability

The data that support the findings of this study are available from the National Board of Health and Welfare (Socialstyrelsen) and the NDR. Restrictions apply to the availability of these data, which were used under licence for the current study and therefore are not publicly available. Data are, however, available from the authors upon reasonable request and with permission of Socialstyrelsen and NDR.

## Abbreviations

DAG: Direct acyclic graph
NDR: National Diabetes Register
NPR: National Patient Register
