# Supplementary material for: Risk factors for Charcot foot development in individuals with diabetes mellitus
Source: Diabetologia. 2024 Sep 13;67(12):2702–10. doi: 10.1007/s00125-024-06271-9 (PMC11604682; doi:10.1007/s00125-024-06271-9)
Supplement: Supplementary file 1 — ESM (PDF 883 KB) [file 125_2024_6271_MOESM1_ESM.pdf]

Electronic supplementary material

Abbreviations

CF Charcot foot

DAG Direct acyclic graph

ESM Table 1

N= number of observations recorded

	Type 1 (N)	Type 2 (N)	Total (N)
Age (years)	1270	2127	3397
Sex	1270	2127	3397
Duration (years)	1270	2127	3397
Weight (kg)	1216	1934	3150
Height (cm)	1200	1913	3113
Body-mass index	1185	1860	3045
BMI	1270	2127	3397
Systolic	1244	2033	3277
Diastolic	1242	2032	3274
Blood pressure	1270	2127	3397
HbA1c (mmol/mol)	1252	2044	3296
Cholesterol (mmol/L)	1125	1809	2934
High-density lipoprotein, HDL (mmol/L)	1086	1713	2799
Triglycerides (mmol/L)	1096	1696	2792
Low-density lipoprotein, LDL (mmol/L)	1102	1677	2779
Creatinine (µmol/L)	1169	1938	3107
Macroalbuminuria	1270	2127	3397
Microalbuminuria	1270	2127	3397
Blood pressure medication	1270	2127	3397
Lipid-lowering medication	1155	1910	3065
Prev. osteoporosis	1270	2127	3397
Diseases of arteries	1270	2127	3397
Prev. PVD	1270	2127	3397
Prev. atherosclerosis	1270	2127	3397
Prev. atherosclerosis extrem.	1270	2127	3397
Acetylsalicylic acid, ASA	1270	2127	3397
Prev. ischaemic heart disease	1270	2127	3397
Prev. stroke	1270	2127	3397
Diabetic retinopathy	1270	2127	3397
Smoking	1270	2127	3397

ESM Table 1: Number of observations recorded in the characteristics of the individuals with type 1 and type 2 diabetes and CF.

ESM Table 2

N = number of observations recorded

	Type 1 without CF (N)	Type 1 with CF (N)	Total (N)
Age (years)	7209	1270	8479
Sex	7209	1270	8479
Duration (years)	7209	1270	8479
Weight (kg)	6241	1216	7457
Height (cm)	5980	1200	7180
Body-mass index	5731	1185	6916
BMI	7209	1270	8479
Systolic	6619	1244	7863
Diastolic	6603	1242	7845
Blood pressure	7209	1270	8479
HbA1c (mmol/mol)	6793	1252	8045
Cholesterol (mmol/L)	5294	1125	6419
High-density lipoprotein, HDL (mmol/L)	4968	1086	6054
Triglycerides (mmol/L)	4959	1096	6055
Low-density lipoprotein, LDL (mmol/L)	4895	1102	5997
Creatinine (μmol/L)	5731	1169	6900
Macroalbuminuria	7209	1270	8479
Microalbuminuria	7209	1270	8479
Blood pressure medication	7209	1270	8479
Lipid-lowering medication	6564	1155	7719
Prev. osteoporosis	7209	1270	8479
Diseases of arteries	7209	1270	8479
Prev. PVD	7209	1270	8479
Prev. atherosclerosis	7209	1270	8479
Prev. atherosclerosis extrem.	7209	1270	8479
Acetylsalicylic acid, ASA	7209	1270	8479
Prev. ischaemic heart disease	7209	1270	8479
Prev. stroke	7209	1270	8479
Diabetic retinopathy	7209	1270	8479
Smoking	7209	1270	8479

ESM Table 2: Number of observations recorded in the characteristics of the matched individuals with or without Charcot foot and type 1 diabetes.

ESM Table 3

N = number of observations recorded

	Type 2 without CF (N)	Type 2 with CF (N)	Total (N)
Age (years)	20,453	2127	22,580
sex	20,453	2127	22,580
Duration (years)	20,453	2127	22,580
Weight (kg)	18,055	1934	19,989
Height (cm)	17,579	1913	19,492
Body-mass index	17,020	1860	18,880
BMI	20,453	2127	22,580
Systolic	18,902	2033	20,935
Diastolic	18,888	2032	20,920
Blood pressure	20,453	2127	22,580
HbA1c (mmol/mol)	19,348	2044	21,392
Cholesterol (mmol/L)	16,355	1809	18,164
High-density lipoprotein, HDL (mmol/L)	15,013	1713	16,726
Triglycerides (mmol/L)	15,050	1696	16,746
Low-density lipoprotein, LDL (mmol/L)	14,737	1677	16,414
Creatinine (µmol/L)	17,886	1938	19,824
Macroalbuminuria	20,453	2127	22,580
Microalbuminuria	20,453	2127	22,580
Blood pressure medication	20,453	2127	22,580
Lipid-lowering medication	18,715	1910	20,625
Prev. osteoporosis	20,453	2127	22,580
Diseases of arteries	20,453	2127	22,580
Prev. PVD	20,453	2127	22,580
Prev. atherosclerosis	20,453	2127	22,580
Prev. atherosclerosis extrem.	20,453	2127	22,580
Acetylsalicylic acid, ASA	20,453	2127	22,580
Prev. ischaemic heart disease	20,453	2127	22,580
Prev. stroke	20,453	2127	22,580
Diabetic retinopathy	20,453	2127	22,580
Smoking	20,453	2127	22,580

ESM Table 3: Number of observations recorded in the characteristics of the matched indiviuals with or without Charcot foot and type 2 diabetes.

ESM Fig. 1

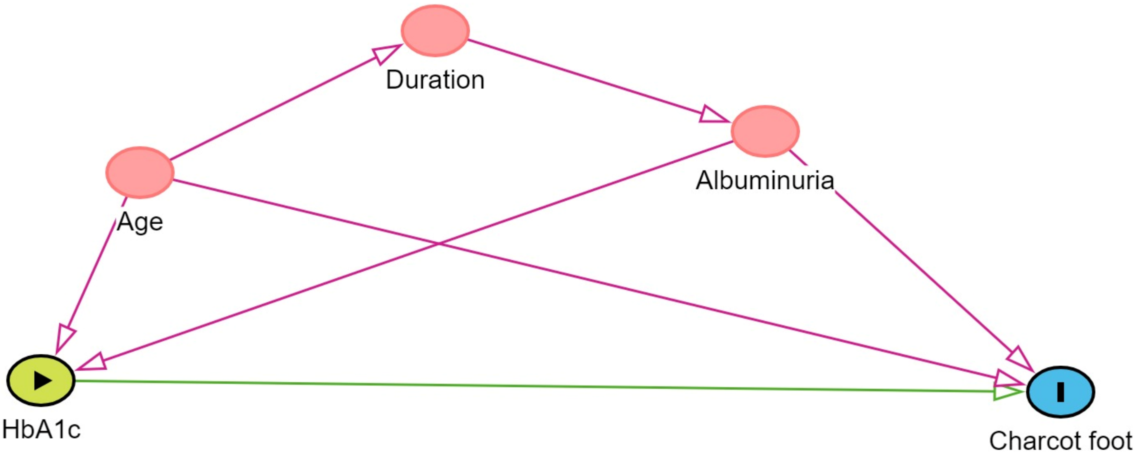

ESM Fig. 1: DAG Type 1 diabetes – HbA1c

ESM Fig. 2

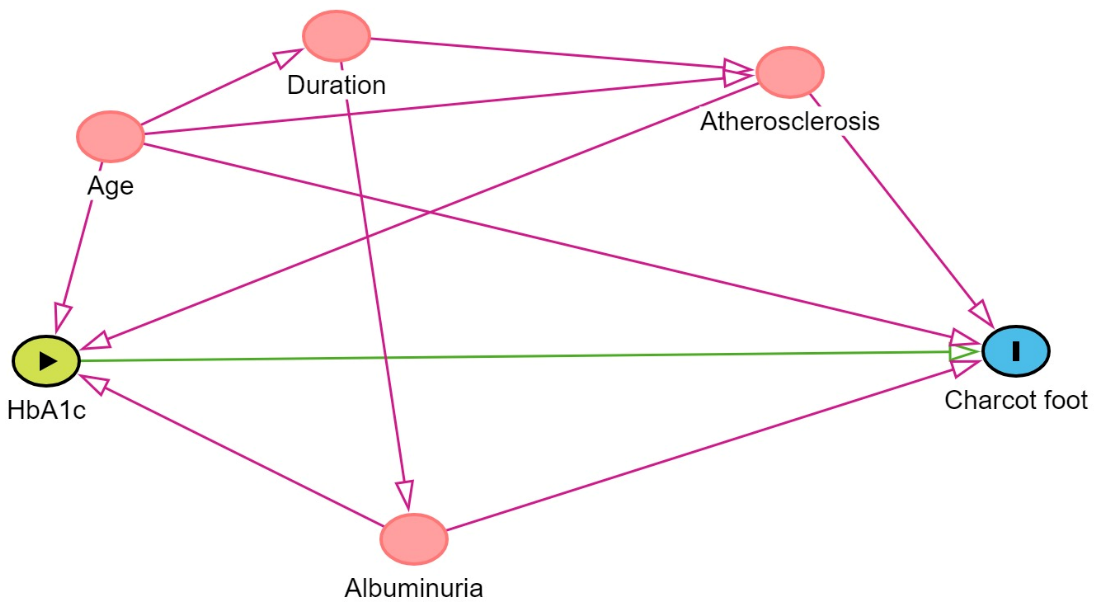

ESM Fig. 2: DAG Type 2 diabetes – HbA1c

ESM Fig. 3

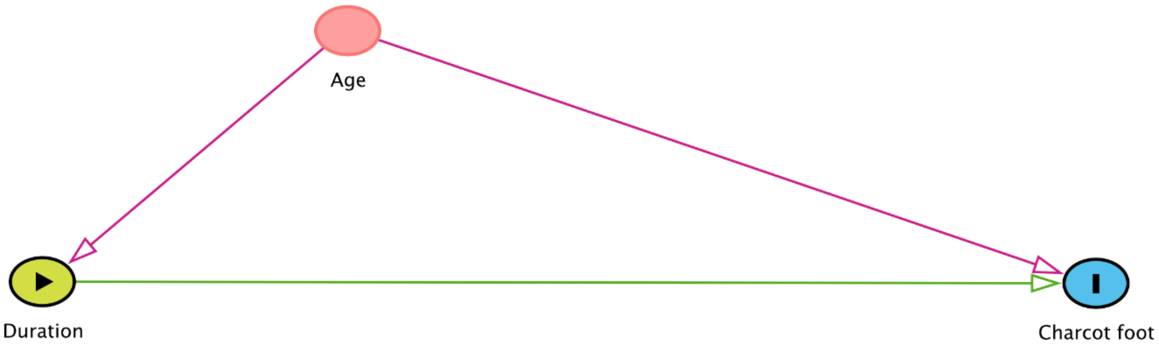

ESM Fig. 3: DAG Type 1 & 2 diabetes – Duration

ESM Fig. 4

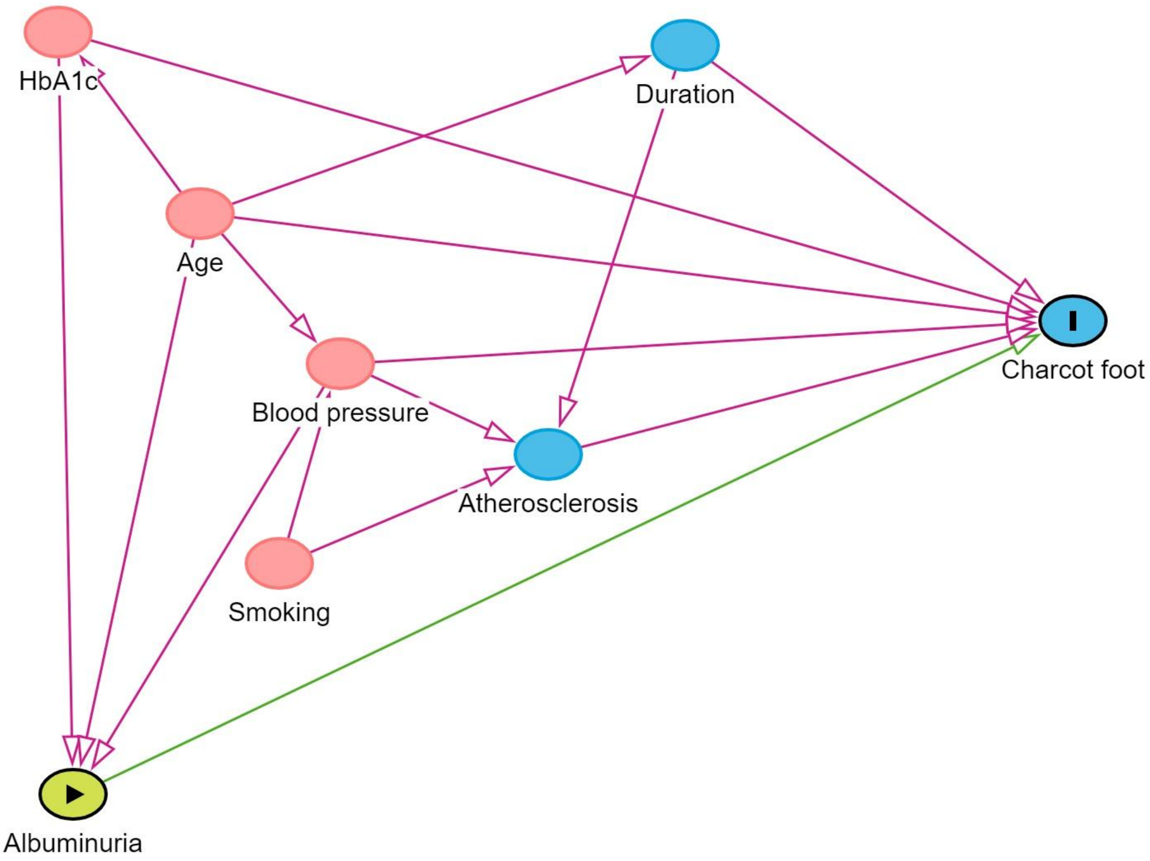

ESM Fig. 4: DAG Type 1 & 2 diabetes – Albuminuria

ESM Fig. 5

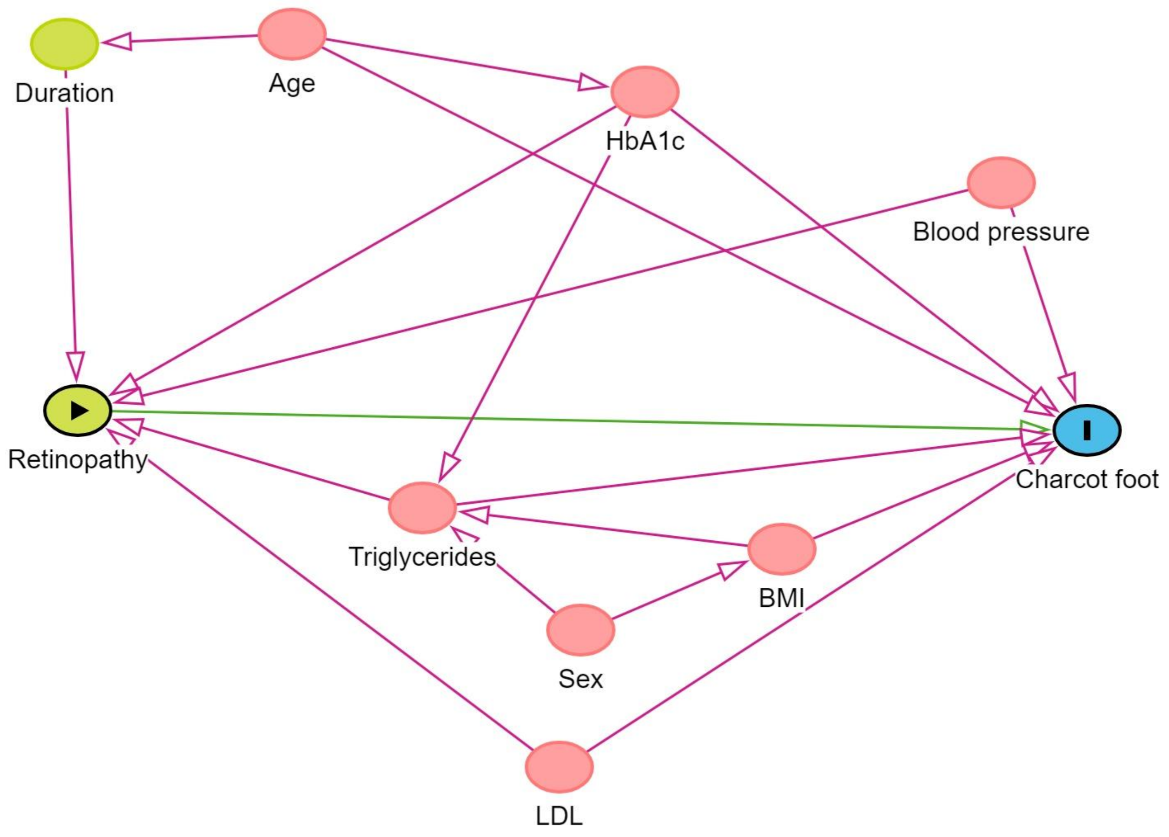

ESM Fig. 5: DAG Type 1 & 2 diabetes – Retinopathy

ESM Fig. 6

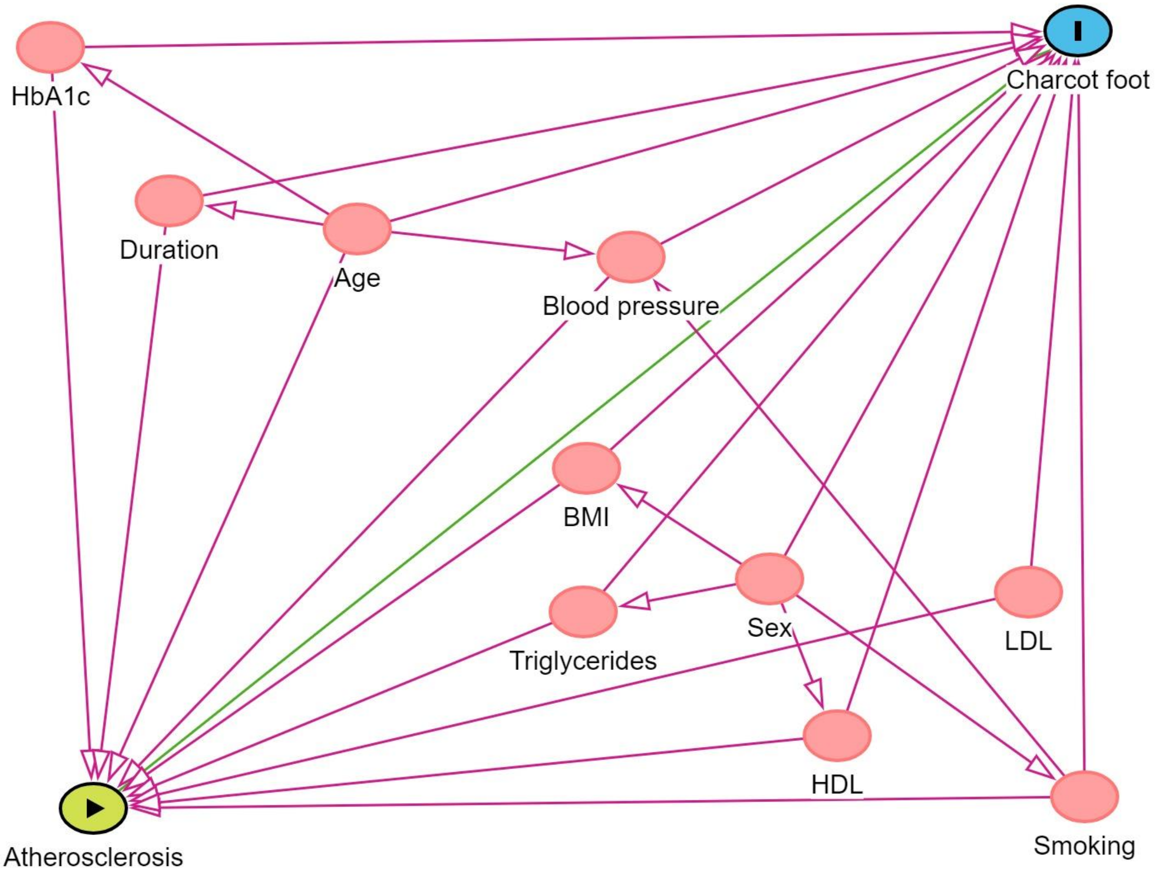

ESM Fig. 6: DAG Type 1 & 2 diabetes - Atherosclerosis
